# Coercion in psychiatry: psychometric validation of the Portuguese Staff Attitudes to Coercion Scale (SACS)

**DOI:** 10.1007/s44192-024-00083-4

**Published:** 2024-08-14

**Authors:** Deborah Oyine Aluh, Diego Diaz-Milanes, Barbara Pedrosa, Manuela Silva, Ugnė Grigaitė, Carolina Rocha Almeida, Maria Ferreira de Almeida Mousinho, Margarida Vieira, Graça Cardoso, José Miguel Caldas-de-Almeida

**Affiliations:** 1 Lisbon Institute of Global Mental Health, Lisbon, Portugal; 2 Comprehensive Health Research Centre (Chrc), NOVA Medical School, NOVA University of Lisbon, Lisbon, Portugal; 3 Department of Clinical Pharmacy and Pharmacy Management, University of Nigeria Nsukka, Nsukka, Nigeria; 4 Department of Quantitative Methods, Universidad Loyola Andalusia, Seville, Spain; 5 Health Research Institute, University of Canberra, Canberra, ACT Australia; 6 Serviço de Psiquiatria E Saúde Mental de Adultos, Hospital de Egas Moniz, Centro Hospitalar de Lisboa Ocidental, Lisbon, Portugal; 7 Department of Mental Health, Unidade Local de Saúde Do Baixo Alentejo, Beja, Portugal; 8 Centro Hospitalar De Vila Nova De Gaia/Espinho, E.P.E.| V. N. Gaia/Espinho Hospital Centre, Vila Nova de Gaia, Portugal

**Keywords:** Staff Attitudes to Coercion Scale (SACS), Psychometric properties, Coercion measures, Validation, Portuguese

## Abstract

**Background:**

There is a growing recognition that staff attitudes toward coercion in mental health care may influence its application. This study presents the psychometric properties of the Portuguese version of the Staff Attitudes towards Coercion Scale (SACS) and describes mental health professionals’ attitudes towards coercion in Portugal.

**Methods:**

Mental health professionals working in five Portuguese mental health services in urban and rural regions of Portugal were invited to complete a questionnaire comprising the SACS and a socio-demographic form. Psychometric analyses including construct validity and internal consistency were carried out using R software.

**Results:**

A total of 91 out of 119 questionnaires completed were valid for analysis. Fifty-seven (62.64%) respondents were female, with an age range of 24 to 69 years (M = 39.33; SD = 11.09). More than half of them were nurses (52.75%, n = 48), and a third were psychiatrists (36.26%, n = 33). A three-factor structure was confirmed and showed the best fit compared to previously proposed models with a cumulative explained variance of 59%. The Portuguese SACS exhibited adequate internal consistency for both the full-scale and subscales. The highest mean score was in the pragmatic attitude domain (20.60; SD = 3.37). A negative correlation was observed between the critical attitude domain and both age and years of experience (p < 0.05).

**Conclusion:**

A three-factor structure was confirmed and showed the best fit compared to previously proposed models. The Portuguese SACS showed excellent psychometric properties and is acceptable for assessing staff attitudes towards coercion.

**Supplementary Information:**

The online version contains supplementary material available at 10.1007/s44192-024-00083-4.

## Introduction

Coercion in mental health care refers to actions such as forced treatments, involuntary procedures employed during treatment or when addressing possible harm an individual may pose to themselves or others. [1] Involuntary admission, outpatient commitment, mechanical restraint, chemical restraint, and seclusion are examples of coercive measures that are usually encoded in law. While these measures are typically intended to help the patient, they can also be employed to safeguard others or, at times, be misused by healthcare professionals, rendering them a morally complex issue [2]. The use of coercive measures to treat people with mental health conditions has come under intense scrutiny and debate in recent times due to the considerable violations of human rights they entail. Several international organizations, including the World Health Organization and the World Psychiatric Association, have strongly advocated for alternatives to coercion in mental health settings. [3, 4] However, to effectively implement these recommendations, it is crucial to gain a thorough understanding of the factors that promote the use of these coercive measures in mental healthcare.

Systematic reviews of the literature have shown that factors such as age, diagnosis, severity of symptoms, race, and ethnicity can impact the likelihood of being subjected to different forms of coercive measures. [5, 6] Organizational factors, such as ward characteristics and the ratio of staff to patients, can also influence the risk of coercion[7] The characteristics of mental health professionals themselves are increasingly recognized as influential in the use of coercion. Several studies have shown links between the gender [7], age [8], qualifications [7, 9, 10] of mental health professionals, and their propensity to use coercive measures. More critical analyses of the clinical decision-making process show that an important, yet frequently overlooked factor implicated in the use of coercive measures is the attitude of the staff towards coercion and how this attitude can be influenced by personality traits, emotions, and previous experiences [11–15]. Attitudes have been defined as ‘learned predispositions to think, feel, and behave in a specific manner toward a certain object’ with three main components: affective, behavioral, and cognitive aspects [16]. The emotional component of attitudes is described by the affective aspect, while sensory responses and persuasions are encompassed by the cognitive component. Actions that align with the attitude are described by the behavioral element [17, 18] According to Fishbein and Ajzen [16], an individual's attitude toward an object mediates every response, and each response can be categorized into one of the three attitude components [16]. However, Azjen’s Theory of Planned Behavior recognizes the intricacy of the attitude-behavior relationship [19] as social interactions and experiences can shape and alter attitudes over time.

Studies conducted over time show that nurses’ perception of coercion has undergone a paradigm shift over time, with a shift from viewing it as therapeutic to perceiving it as a safety measure [8] In the safety paradigm, coercive measures are considered a last resort, and there is a preference for the least intrusive intervention. This has resulted in a dilemma for nurses, as they believe that coercion is necessary, but its use can evoke negative emotions. A systematic review of studies on staff attitudes towards coercion revealed that staff consider coercive measures to be essential tools in mental health care for ensuring safety and providing necessary care for individuals, highlighting their concern with safety issues [20] In the United States, psychiatrists were more likely to support involuntary hospitalization if they believed in meeting basic needs and less likely if they believed in respecting the right to refuse treatment or the unpredictability of the future [21]. In Switzerland, although a significant proportion of mental health professionals viewed coercion as a violation of basic human rights, they believed that coercion was necessary and beneficial for psychiatric patients [22] Before we can effectively modify the attitudes of mental health professionals towards the use of coercion, it is crucial to gain a comprehensive understanding of those attitudes. This understanding and modification serve as a critical component in interventions aimed at reducing coercion in mental health care.

A psychometrically sound instrument is needed for evaluating the attitudes of mental health professionals toward coercion. The present study sought to evaluate the Staff Attitude toward Coercion Scale (SACS) as such an instrument. The SACS is a 15-item self-reported instrument developed by Husum et al. in [23] among a Norwegian sample of mental health professionals. [23] It is comprised of three subdomains: a critical attitude toward coercion, a pragmatic attitude toward coercion, and a positive attitude toward coercion. The SACS has become one of the most widely and internationally used instruments for evaluating staff attitudes towards coercion, having been translated into several languages, including Arabic [24], Chinese [25], German [26], Japanese [27], Italian [28] and Polish [29], and administered more than 5000 times. Portuguese is the sixth most natively spoken language, across eleven countries and three continents in the world. Not many studies have assessed the attitudes of mental health professionals towards coercion among Portuguese-speaking populations, and the SACS has never been used or validated in a Portuguese context. The subject of staff attitudes towards coercion has not received attention in Portugal, nor in other Portuguese-speaking countries, and it seems logical to expect that validating a Portuguese version of the SACS could steer attention to this area of research among Portuguese-speaking countries. In addition, the psychometric literature on SACS has highlighted some doubts about the factor structure and cross-cultural validity of the SACS. The present study aimed to explore the psychometric properties, in terms of validity and reliability, of the SACS Portuguese version and compare the results with those reported in previous validation studies presented in the literature. The study also describes the attitudes of mental health professionals in Portugal towards coercion, and associated factors.

## Materials and methods

### Study design and participants

### Study procedure

The original questionnaire was validated among a sample of Norwegian mental health professionals and was reported to have acceptable psychometric properties. [23] The three subscales demonstrated Cronbach Alpha coefficients of 0.70, 0.73, and 0.69, respectively, while the overall scale, encompassing all 15 items, had a Cronbach Alpha coefficient of 0.78. To conduct the cross-cultural adaptation of the SACS to Portuguese, the following steps were taken:

Firstly, the English version of the SACS as provided by the original developers [23] was translated to Portuguese by one of the authors, who is a native Portuguese speaker, and then reviewed by two co-authors who are native Portuguese speakers. This version was piloted among five mental health professionals to check for clarity and understanding. As the responses were favorable, the Portuguese version of the SACS was then back translated to English by an independent translator who was not a member of the research team. The back translated questionnaire was additionally reviewed by the research team to ensure that meaning was retained. No changes were required at this stage. The final version of the paper-based questionnaire was administered in person to all staff members who had worked for a minimum of 6 months in each of the mental health services who were willing to participate in the study, between February 2022 and February 2023.

### Study instrument

The study instrument comprised two parts, a short demographic form, and the SACS. The SACS is a 15-item questionnaire (SACS) that assesses professionals’ feelings and attitudes towards coercion on a 5-point Likert scale from strongly disagree (scored 1) to strongly agree (scored 5) with three dimensions namely, Coercion as offending (critical attitude)—the view of coercion as offensive towards patients; Coercion as care and security (pragmatic attitude)—the view of coercion as needed for care and security. The use of coercion is deemed necessary for safety and security reasons in this position, despite not being regarded as a positive or desirable approach. The third dimension is Coercion as treatment (positive attitude)—the view of coercion as a treatment intervention, comprising items that hold a favorable perspective towards employing coercion. When utilizing the 15 items as a complete scale, the items within the first subscale (critical attitude) are reversed to indicate a higher score, indicating a more favorable attitude towards the implementation of coercion.

### Data analysis

The software used was R (Version 4.1.3) [30] for data processing and statistical analyses by implementing the packages: dplyr (Version 1.1.0), psych (Version 2.2.3), semTools (Version 0.5.6), semPlot (Version 1.1.5) and ggplot2 (Version 3.4.1).

Descriptive statistics of the sample were first conducted to obtain the actual number of valid responses for further analysis and their characteristics for posterior comparative analysis. Next, univariate, and multivariate descriptive statistics were performed on each item of the instrument to assess and select the optimal correlation matrix for the following analysis. Items that expressed negative opinions about coercion were conversely recoded to maintain their coherence with the rest of the instrument. An exploratory factor analysis (EFA) was then conducted. Before this, the Kaiser–Meyer–Olkin (KMO) test of sample adequacy and Bartlett’s test of sphericity and determinant of the matrix were conducted. To select the most reliable and parsimonious factorial structure, optimal coordinates, parallel analysis, and Kaiser criterion methods were used to calculate the number of factors to be extracted. Subsequently, the factor solution obtained was tested in confirmatory factor analysis (CFA). The fit measures applied were the χ^2^ statistic, degrees of freedom, and p-value of χ^2^, the comparative fit index (CFI), the Tucker-Lewis index (TLI), the Non-Normed Fit Index (NNFI) and the root mean square error of approximation (RMSEA). Cut-off criteria were as follows: ≤ 3 for the χ2/df ratio; ≥ 0.90 for the CFI and TLI; and ≤ 0.08 for the RMSEA [31] Internal consistency analyses were then performed through ordinal alpha, Guttman split-half, McDonald’s omega, and the correlations between each item and the total score of the scale. Percent attenuation of Cronbach’s alpha was conducted for the full scale and each subscale [32] To explore the associations between staff characteristics and their attitudes towards coercion, the total score and every subscale score were compared with participant characteristics by using the Pearson correlation coefficient, Mann–Whitney-Wilcoxon test, or Kruskall-Wallis test, with Bonferroni correction for pairwise comparisons, as required.

### Ethical considerations

The study's procedures followed the ethical standards outlined by the relevant national and institutional committees for human experimentation, including the 2008 revised Helsinki Declaration of 1975. The study was approved by the Research Ethics Committee of the university (100/2021/CEFCM), as well as the ethics committees of all participating hospitals. Written informed consent was obtained from all the study participants.

## Results

### Characteristics of the sample

A total of 119 mental health professionals from different hospitals agreed to complete the survey. A total of 14 respondents did not complete any of the items of the  SACS, and 14 did not provide their professional or socio-demographic data. Thus, the final study sample comprised 91 participants, of whom 62.64% were female and 37.36% were male, with an age range of 24 to 69 years (M = 39.33; SD = 11.09). More than half of the participants were nurses (52.75%, n = 48), and a third were psychiatrists (36.26%, n = 33). More than three-quarters (82.42%, n = 75) had applied a coercive intervention within the preceding 6 months, and less than half (42.86%, n = 39) had received any training on the use of coercion (Table 1).

**Table 1:** Socio-demographic characteristics of the sample

Variable	N (%)
Gender	
Male	34 (37.36)
Female	57 (62.64)
Age group	
20–29	18 (19.78)
30–39	37 (40.66)
40–49	18 (19.78)
50–59	12 (13.19)
60 or more	6 (6.59)
Profession	
Psychiatrist	33 (36.26)
Nurse	48 (52.75)
Occupational therapist	4 (4.4)
Social worker	3 (3.3)
Other	3 (3.3)
Place of principal activity	
General hospital	72 (79.12)
Psychiatric hospitals	12 (13.19)
Community mental health team	7 (7.69)
Years of experience	
Less 5 years	16 (17.58)
5–9 years	17 (18.68)
10–14 years	19 (20.88)
15–19 years	17 (18.68)
20 or more	22 (24.18)
Type of setting	
Urban	78 (85.71)
Rural	13 (14.29)
Involvement in coercion in preceding 6 months	
Yes	75 (82.42)
No	16 (17.58)
Specialized training on coercion	
Yes	39 (42.86)
No	52 (57.14)
Relative with a mental health condition	
Yes	48 (52.75)
No	43 (47.25)

### Descriptive analysis of SACS items

Table 2 shows the means, standard deviations, minimum and maximum score, skewness, and kurtosis of the 15-items scale. Although the items showed a skew in the range of − 2 and + 2, the kurtosis value exceeded the limit for items 1,5 and 9, indicating they cannot be considered univariate normally distributed. Furthermore, the tests of multivariate skewness (χ2(680) = 1192.810, p < 0.001) and multivariate kurtosis (z = 6.555, p < 0.001) were both statistically significant, indicating that the data did not follow a multivariate normal distribution and that the Polychoric correlation matrix was the most suitable for factorial analysis and reliability calculation. The highest mean score was in the pragmatic attitude domain (Coercion as Care and security) (Table 2).

**Table 2:** Descriptive statistics of the SACS items and domains

Item		Mean	SD	Range (min–max)	Skew	Kurtosis
1	Use of coercion is necessary as protection in dangerous situations	4.297	0.850	1–5	− 1.455	2.361
2	For security reasons coercion must sometimes be used	4.154	0.842	1–5	− 1.173	1.720
3*	Use of coercion can harm the therapeutic relationship	2.000	1.022	1–5	− 0.927	0.244
4*	Use of coercion is a declaration of failure on the part of the mental health services	3.791	1.006	1–5	0.488	− 0.58
5	Coercion may represent care and protection	4.000	0.894	1–5	− 1.474	2.769
6	More coercion should be used in treatment	2.253	1.039	1–5	0.782	0.248
7	Coercion may prevent the development of a dangerous situation	4.000	0.931	1–5	− 0.981	0.599
8*	Coercion violates the patient’s integrity	3.187	1.115	1–5	0.272	− 0.800
9	For severely ill patients, coercion may represent safety	4.154	0.868	1–5	− 1.505	2.946
10	Patients without insight require use of coercion	2.451	1.014	1–5	0.291	− 0.829
11	Use of coercion is necessary toward dangerous and aggressive patients	3.670	1.023	1–5	− 0.428	− 0.722
12	Regressive patients require use of coercion	2.538	0.821	1–5	0.057	− 0.003
13*	Too much coercion is used in treatment	3.187	1.173	1–5	0.320	− 0.886
14*	Scarce resources lead to more use of coercion	2.385	1.123	1–5	− 0.518	− 0.560
15*	Coercion could have been much reduced, giving more time and personal contact	2.143	1.081	1–5	− 0.711	− 0.396

Mean scores of subscales						
Scale	Score	Mean	SD	Min	Max	Mean normalization from 1 to 10
Total	Global	48.20879	8.614608	23	65	6.40188357
Care and security	F1	20.6044	3.372698	10	25	7.36264
Offending (reverse)	F2—Reverse	16.69231	4.699154	8	26	5.346155
Offending	F2	19.30769	4.699154	10	28	5.653845
Treatment	F3	10.91209	3.017167	4	20	4.88805063

^*^ These items were conversely recoded

### Reliability based on internal consistency measures

All items have corrected Item-Total correlations ranging from 0.45 (“11. Use of coercion is necessary toward dangerous and aggressive patients”) to 0.77 (“1. Use of coercion is necessary as protection in dangerous situations”).

The 15-item scale showed an ordinal alpha of 0.89 (95% CI 0.78–0.95). Cronbach’s alpha for the same items was 0.86, indicating an attenuation effect of -3.19%. In the case of the first factor, the ordinal alpha is 0.88 (95% CI 0.57–0.99), for the second factor is 0.85 (95% CI 0.52–0.98), and for the third factor is 0.83 (95% CI 0.15–0.99), while the Cronbach’s alphas are 0.83, 0.81 and 0.77, respectively (attenuation effect of − 6.52%, − 3.7% and − 7.25%). In the case of the third factor, eliminating item 11 would improve the alpha value by 0.03, but maintain similar 95% CI. Nevertheless, eliminating any item for the full scale and the other sub-scales did not improve their alphas.

Regarding the McDonald’s omega, the full scale showed a value of 0.88 while the first, second and third factors were 0.84, 0.83 and 0.81, respectively.

Finally, the Guttman Coefficient obtained from the split half test showed a value on average of 0.89, with a minimum of 0.67 and a maximum of 0.96, for the full scale. In reference to the sub-scales, the first factor showed a 0.86 (min: 0.82; max: 0.88), the second a 0.85 (min: 0.79; max: 0.92), and the third 0.83 (min: 0.82, max: 0.85).

### Evidence of validity based on the internal structure

The results of the Kaiser–Meyer–Olkin test (KMO = 0.77), Bartlett’s sphericity test (χ2(105) = 820.633; p < 0.001) and the correlation matrix determinant (< 0.001) showed that the responses to the scale could be considered adequate for a dimension reduction.

### Exploratory factor analysis (EFA)

An EFA was conducted using the Diagonally Weighted Least Squares (DWLS) extraction method on the polychoric correlation. Regarding the number of factors, the three methods applied (optimal coordinates, parallel analysis, and Kaiser criterion methods) supported the extraction of three factors. An oblique rotation (Promax method) was selected. The three-dimension structure had a cumulative explained variance of 59% (Factor 1 = 22%; Factor 2 = 21%; Factor 3 = 16%). Table 3 shows the factor loadings of each item after eliminating those below 0.40. Regarding the communalities of the items, all values exceeded 0.30 with the lowest being item 11 with 0.35 (“Use of coercion is necessary toward dangerous and aggressive patients”) (Table 3).

**Table 3:** Standardized loadings (pattern matrix) on the polychoric matrix using diagonally weighted least squares

Item*	F1	F2	F3	h2	u2
1	0.80			0.77	0.23
2	0.85			0.70	0.30
3		0.67		0.46	0.54
4		0.48		0.37	0.63
5	0.77			0.63	0.37
6			0.52	0.60	0.40
7	0.67			0.51	0.49
8		0.61		0.44	0.56
9	0.90			0.69	0.31
10			0.90	0.78	0.22
11			0.54	0.35	0.65
12			0.87	0.79	0.21
13		0.79		0.57	0.43
14		0.85		0.64	0.36
15		0.63		0.60	0.40

^*^F = Number of the factors; h2 = communality of the item; u2 = uniqueness of the item; Weights lower than 0.40 are removed

A Confirmatory Factor Analysis was thus needed due to the differences between the solution obtained and the models from previous studies.

### Confirmatory factor analysis (CFA)

**Fig. 1:**
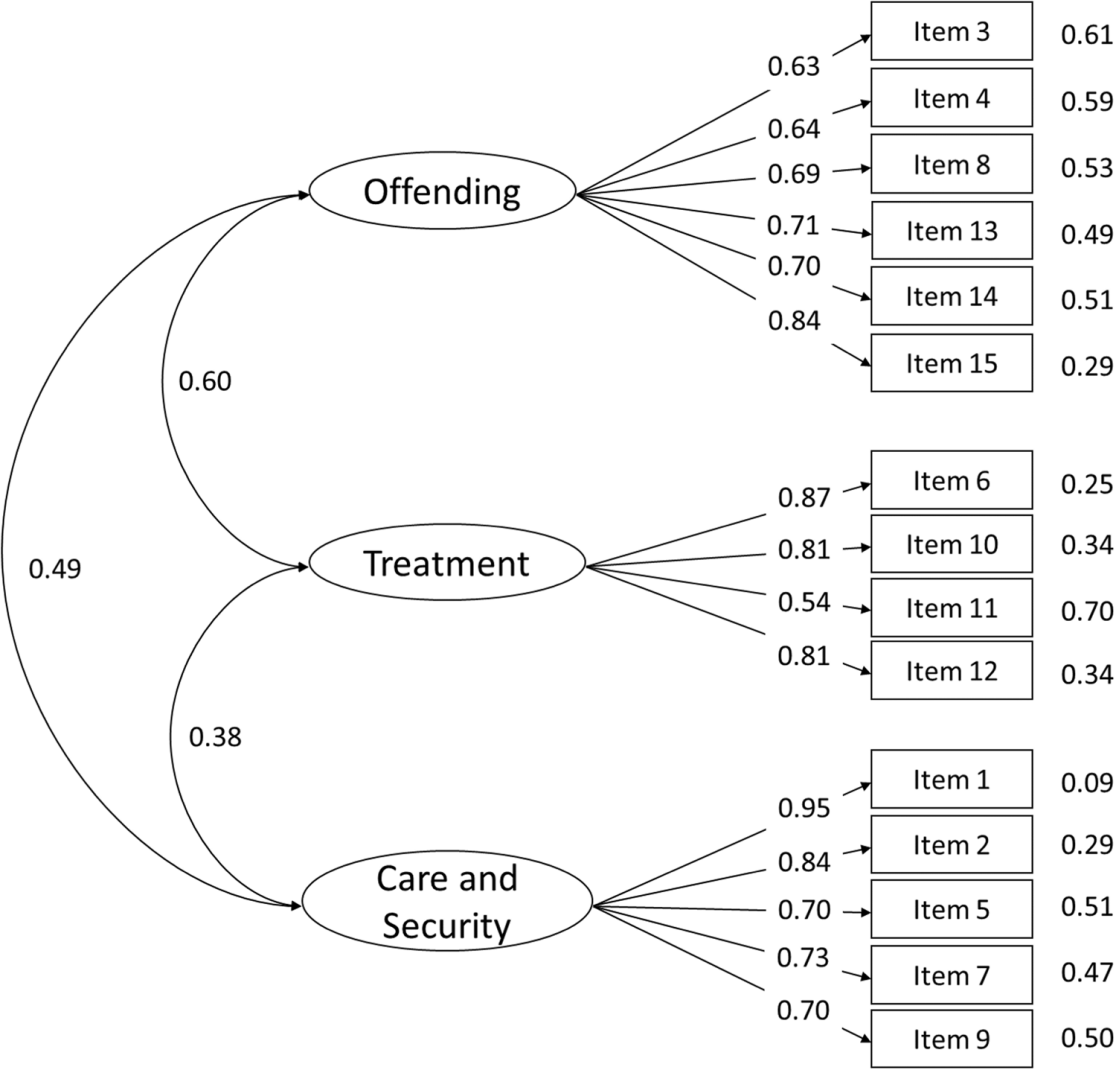
Path diagram of the three-factor structure obtained showing the estimated three-factor model with the factor loadings of each item and their residuals. After the negative wording items were conversely recoded, the correlation among factors changed from 0.38 to 0.60

To evaluate the suitability of four models using DWLS, a set of CFAs were conducted. The German version’s one-factor model [26] did not demonstrate a satisfactory fit, while the original English version’s three-factor model and the Japanese four-factor model [23, 27]exhibited acceptable fits based on most of the indices but not regarding the RMSEA. On the other hand, the current study’s three-factor structure (Fig. 1) demonstrated the best fit. More details can be found in Table 4.

**Table 4:** Fit indexes for models tested

Model*	χ2	Df	*p-value*	CFI	TLI	NNFI	RMSEA
Three-factors	134.727	87	0.001	0.983	0.979	0.979	0.078
Original three-factors	155.915	87	< 0.001	0.975	0.970	0.970	0.094
One-factor	433.154	90	< 0.001	0.874	0.853	0.853	0.206
Four-factor	161.397	84	< 0.001	0.972	0.965	0.965	0.101

^*^χ2 Chi-Square statistic, *df* degrees of freedom, *CFI* Comparative Fit Index, *TLI* Tucker Lewis Index, *NNFI* Non-Normed Fit Index, *RMSEA* Root Mean Square Error of Approximation

### Bivariate scale score comparisons

No significant differences were observed among socio-demographic characteristics of the staff when the full scale was considered, significant differences were only observed in the case of the first and second factors. For the Coercion as care and security dimension, significant differences were found (W = 408, *p* = 0.044) between professionals who had applied coercive measures within the preceding 6 months (Median = 21) and those who had not (Median = 20). For the Coercion as treatment dimension, some differences were found regarding the location of the services (W = 328.5, *p* = 0.042), staff of services in urban areas had higher scores in this domain (Median = 21) compared to the staff working in the service located in the rural area (Median = 20). For the coercion as offending dimension, there was a significant negative correlation with age (r = − 0.230, *p* = 0.028) and years of experience (r = − 0.229, *p* = 0.029), respectively.

## Discussion

Significant changes in the attitudes of mental health staff are necessary for the successful implementation of strategies to reduce coercion in mental health care [33]. Understanding the attitudes of staff towards coercion is recognized as a key step in formulating interventions that can reduce coercion in each setting. To the best of our knowledge, this is the first study to develop and structurally validate the Portuguese version of the SACS. Perhaps more importantly, this is the first attempt to assess the fit of previously proposed models. A notable strength of this psychometric validation is that a polychoric correlation matrix was rationally chosen for factorial analysis and reliability calculation because a prior normality test showed that the items did not follow a univariate or multivariate normal distribution. Previous studies that validated the SACS did not mention the matrix used, mostly because Pearson product-moment correlation is assumed by most software. Since the SACS employs a Likert scale to assess responses and generates ordinal data, utilizing polychoric correlations to obtain solutions offers a more precise replication of the measurement model employed to produce such data [34]. The results from our study support a new three-factor model for SACS, slightly different from the one obtained from the original version. Given the limited sample size, additional research with larger populations is necessary to confirm these findings. Differences in the pragmatic and critical attitudes dimensions were observed for socio-demographic characteristics such as age, years of experience, location of service, and application of coercive measures in the preceding 6 months.

To show that the data collected over time is stable and consistent, it is necessary to use standardized questionnaires. The use of reliable instruments ensures that results can be replicated when the same population is surveyed at different times, and even when individuals from diverse cultural backgrounds are surveyed [35]. Although the Chi-square test was significant, it is important to consider the results of the other indicators as well, as this indicator can be influenced by sample size [36]. The three-factor solution obtained in the present study showed the best fit and good internal consistency from three different methods. The attenuation effect was small for all the cases, and removing any of the items did not show a significant increase in reliability. Item 11 (Use of coercion is necessary toward dangerous and aggressive patients) loaded on the positive attitude dimension in our model rather than the pragmatic attitude dimension as proposed in the original validation study. However, both the original study and the Japanese study reported that this item loaded on two dimensions (pragmatic and positive attitude dimensions) [23, 27]. Although it is typical for empirical data to exhibit cross-loadings [37], it could also indicate that the safety paradigm conveyed by pragmatic attitudes towards coercion is not entirely distinct from the therapeutic paradigm conveyed by positive attitudes towards coercion.

The instrument also showed good reliability based on its internal consistency indices (Ordinal Alpha, McDonald’s Omega, and split-half Gutman coefficients). Adequate reliability scores for the whole scale and the three different subscales were obtained, implying that it is a reliable measure for use among Portuguese mental health professionals. This is consistent with previous studies among German, Japanese and Norwegian professionals [23, 26, 27]. Improved alpha values following the removal of an item may suggest that certain items are not well-suited for the instrument. In the case of the third factor, eliminating item 11 (use of coercion is necessary toward dangerous and aggressive patients) slightly improved the alpha value. Despite the increase, the coefficient value remained within the reliability estimate’s 95% CI. As a result, the psychometric indicators were not significantly impacted, which may have resulted in a less accurate representation of the item's contents.

In the current study, the mental health professionals in Portugal showed the highest scores on the SACS “Coercion as Care and Security subscale,” consistent with the finding among Norwegian mental health professionals [23, 38] but differing from German mental health professionals who scored highest in the “Coercion as Offending subscale” [14]. Differences in attitudes towards coercion have been observed based on gender [13] years of experience, [13] and occupational groups [14, 39]. In this study, no significant differences were observed for different socio-demographic characteristics when the entire scale was considered, however, some differences were observed for subscales. Mental health professionals who had applied coercive interventions in the previous 6 months had significantly higher scores in the pragmatic attitude subscale compared to those who had not. In addition, some differences were found regarding the location of the services. The staff of services located in urban areas had a more positive attitude towards coercion (“Coercion as treatment subscale”) than the staff working in the service located in the rural area. This could be because of the higher demand and pressure on mental health services in urban areas, and the higher chances of meeting service users with different cultural backgrounds. The literature on the association between years of experience and attitudes toward coercion is not consistent. Some studies have indicated that individuals with fewer years of work experience tend to exhibit more positive attitudes towards coercion [11], whereas others have reported the opposite trend [33]. A significant negative association was observed between the critical attitude dimension (“Coercion as offending subscale”) and age and years of experience, meaning that the older the professionals were and the longer they worked in a service, the less critical attitude they had toward coercion. While the small sample size in this study limits the conclusions that can be drawn, the varied attitudes observed suggests that a one-size-fits-all approach to changing staff attitudes towards coercion may not be effective. Instead, tailored interventions targeting specific socio-demographic and professional profiles are likely to be more successful.

## Strengths and limitations

This is the first study conducted to investigate the factor structure of staff attitudes towards coercion in mental health care in a Portuguese-speaking population. It is also the first to assess the fit of the model, comparing it with previously reported models from previous studies. Unfortunately, due to its cross-sectional design, this study could not explore the psychometric properties of the SACS over time, thus being unable to assess the test–retest reliability. Additionally, the small sample size did not allow us to perform an invariance structure analysis, which can be part of further studies. Finally, only one measure of staff attitude towards coercion was used in this study (i.e., SACS), which prevents us from evaluating concurrent validity and structural validation across other instruments. As the decision-making process is influenced by factors beyond those captured by the SACS, including emotions and personality traits, future studies could enhance current understanding by utilizing the SACS in combination with other instruments to capture a more comprehensive range of variables influential in shaping attitudes towards coercion.

 In conclusion, the Portuguese SACS showed adequate psychometric properties and a new three-factor structure, which showed the best fit when compared to previously proposed models. The results suggest that the Portuguese version of SACS can be used effectively to evaluate the attitudes of staff towards coercion. It may identify variations in staff attitudes based on their sociodemographic and professional characteristics, enabling targeted interventions for staff. The research outcome will enhance the precise evaluation of attitudes regarding coercion and promote the instrument's cross-cultural applicability. The mental health professionals in Portugal showed the highest scores on the pragmatic attitude domain, significant negative association were observed between the critical attitude domain and age and years of experience.

### Supplementary Information

Below is the link to the electronic supplementary material.

Supplementary Material 1.

## Data Availability

The anonymized data that support the findings of this study are not openly available due to reasons of confidentiality  and are available from the corresponding author upon reasonable request. Data are located in controlled access data storage at the Lisbon Institute of Global Mental Health.
