# Supplementary material for: Coercion in psychiatry: psychometric validation of the Portuguese Staff Attitudes to Coercion Scale (SACS)
Source: Discov Ment Health. 2024 Aug 14;4(1):27. doi: 10.1007/s44192-024-00083-4 (PMC11324637; doi:10.1007/s44192-024-00083-4)
Supplement: Supplementary file 1 — Supplementary Material 1. [file 44192_2024_83_MOESM1_ESM.docx]

Appendix

The English version of the SACS may be obtained from the original developers of the instrument in Norway (Husum et al., 2008).

Reference: Husum, T. L., Finset, A., & Ruud, T. (2008). The Staff Attitude to Coercion Scale (SACS): reliability, validity and feasibility. *International Journal of Law and Psychiatry*, *31*(5), 417–422. https://doi.org/10.1016/J.IJLP.2008.08.002

**Sentimentos e atitudes dos profissionais em relação à coerção**

Coerção refere-se a hospitalização involuntária, tratamento compulsivo, medicação forçada, isolamento, unidades de internamento à porta fechada e imobilização.

1 = Discordo totalmente; 2 = Discordo; 3 = Não concordo nem discordo; 4 = Concordo; 5 = Concordo completamente

| S/N |  | 1 | 2 | 3 | 4 | 5 |
| --- | --- | --- | --- | --- | --- | --- |
| 1 | O uso de coerção é necessário como forma de protecção em situações perigosas |  |  |  |  |  |
| 2 | Devido a questões de segurança, a coerção deve ser por vezes usada |  |  |  |  |  |
| 3 | O uso de coerção pode ter um impacto negativo na relação terapêutica |  |  |  |  |  |
| 4 | O uso de coerção é uma declaração de falha por parte dos serviços de saúde mental |  |  |  |  |  |
| 5 | A coerção pode representar cuidado e protecção |  |  |  |  |  |
| 6 | A coerção deveria ser mais usada no tratamento |  |  |  |  |  |
| 7 | A coerção pode prevenir o desenvolvimento de uma situação perigosa |  |  |  |  |  |
| 8 | A coerção viola a integridade do utente |  |  |  |  |  |
| 9 | Para utentes gravemente doentes, a coerção pode representar segurança |  |  |  |  |  |
| 10 | Os doentes sem crítica para a doença requerem o uso de coerção |  |  |  |  |  |
| 11 | O uso de coerção é necessário em utentes perigosos e agressivos |  |  |  |  |  |
| 12 | Utentes regressivos necessitam de uso de coerção |  |  |  |  |  |
| 13 | Demasiada coerção é usada no tratamento |  |  |  |  |  |
| 14 | A escassez de recursos leva a maior uso de coerção |  |  |  |  |  |
| 15 | A coerção poderia ser bastante reduzida através de mais tempo e contacto pessoal |  |  |  |  |  |
